# Intentional Devitalization of the Dental Pulp

**Published:** 1889-08

**Authors:** William H. Trueman

**Affiliations:** Philadelphia, Pa.


### International Dental Journal.

Vol. X. August, 1889. No. 8.

### Original Communications.[1]

### INTENTIONAL DEVITALIZATION OF THE DENTAL PULP.[2]

BY WILLIAM H. TRUEMAN, D.D.S., PHILADELPHIA, PA.

Whether it is ever right, or under what conditions we are justified, in devitalizing a dental pulp, we do not propose at this time to consider. In preparing this paper my purpose has been to call attention to some matters not usually referred to by writers upon this subject,—matters that in my judgment have much to do with the final result of the operation. I shall therefore omit, as far as may be, all generally accepted theories, presuming that you are all perfectly familiar with them.

The difference between the condition of a tooth and its surroundings where devitalization has been the result of progressive pathological changes, and a tooth where devitalization is the desired result of therapeutic treatment, is so great that I have considered it worthy of separate and distinct consideration. In the first instance, our most earnest efforts are directed to combat pathological conditions, either active or passive, already existing; in the

[1] The editor and publishers are not responsible for the views of authors of papers published in this department, nor for any claim to novelty, or otherwise, that may be made by them. No papers will be received for this department that have appeared in any other journal published in this country. The journal is issued promptly on the 15th of the month.

[2] Read at the nineteenth annual session of the New Jersey State Dental Society, Asbury Park, July 17–19, 1889.

latter, while we may not in all cases safely say that no pathological lesions are present, our efforts are mainly to prevent irritation, and to place the tooth in a condition as nearly normal as the mutilation it has suffered will permit.

Dr. H. C. Register, of Philadelphia, in a recently-reported discussion upon root-filling, has concisely and clearly defined the three conditions usually met with in treating pulpless teeth.[1]

I have been impressed with the simplicity and clearness of the classification there given, which is very briefly as follows:

First, devitalized teeth, in which the pulp has been destroyed by intent; second, devitalized teeth, in which the pulp has died without being exposed to atmospheric influences, and has become putrescent; third, devitalized teeth, in which the pulp has been from the onset open to atmospheric influence, and is not only putrescent, but complicated with various septic conditions.

We might readily, as the text-books usually do, complicate this classification. I question, however, if it could be practically improved. It is a natural one. It indicates progressive pathological conditions, and suggests the needed treatment. A little thought will show the wisdom of some such classification, the separate and distinct treatment each class should receive, and with equal force that the confusion and apparent contradictions so noticeable in many discussions upon the treatment of devitalized teeth have arisen mainly from failure to appreciate these distinctively differing conditions.

I propose to confine my remarks to that class to which Dr. Register first refers,—viz., teeth that have been intentionally devitalized, the pulps being removed and the pulp-canals filled before any degenerative action has resulted from their devitalization; my purpose being to call attention to lesions that often exist, and which are the frequent cause of ultimate failure, notwithstanding the complete removal of the devitalized tissue and the thorough filling of the space it occupied.

We will now proceed to consider—only so far, however, as to trace the influence they may have upon the final result of the operation—some of these primary lesions, presuming that they have been sufficiently serious to justify devitalization of the pulp.

It is seldom that we are called upon to devitalize a tooth that is quite normal. Except preparatory to constructing a piece of bridge-work, I can think of no other instance in which it is likely

---

[1] INTERNATIONAL DENTAL JOURNAL, May, 1888, vol. ix. p. 290.

to be done. There is usually, if not always, some previous lesion, such as inflammation, slight or extensive exposure of the pulp, extensive caries without pulp exposure; devitalization being deemed expedient to secure reliable anchorage for the filling, or preparatory to inserting a crown, etc. In all such cases there has been more or less active pulp irritation, that may or may not prove detrimental to the future usefulness and comfort of the tooth.

Devitalization may be necessary to relieve pain caused by ossification of the pulp, encroachment upon the pulp by secondary dentine, etc. These conditions, except that they may render more difficult the complete removal of the devitalized tissue, are not usually serious complications. The formation within the substance of the pulp of nodulary dentine—a condition that, when requiring treatment at all, imperatively calls for devitalization—is a much more serious matter. These cases, when demanding treatment, are at times most obscure and unsatisfactory, especially when they exist in teeth otherwise normal. There is usually long-continued neuralgic pain extremely difficult to locate, the tooth in fault rarely giving any sign until the irritation has progressed so far that removal of the cause in many cases fails to effect a satisfactory cure. The tooth may be rendered, by devitalization and proper after-treatment, passably comfortable for a time, and its usefulness measurably restored, but there is apt to remain a soreness upon pressure and a general indefinite feeling of discomfort that eventually leads to its extraction. Nodulary dentine does not always prove so serious; indeed, it is seldom that we are called upon to treat it as a distinct lesion. It is found quite frequently in treating pulp exposure, where it does not seem to have been of itself at all serious, the previous pain and discomfort being no greater than might reasonably have been expected from the carious condition of the tooth. In such cases, except so far as its presence interferes with the action of the destructive agent and the removal of the devitalized tissue, it has seemed to have been a matter of no moment. Why it should in some cases prove to be so serious and in others not, I am unable to explain.

Considering the final result of treatment, I now pass to a more serious condition,—namely, cases where there has been active inflammation of the pulp. In these cases, no matter what may have been the causes of the inflammation, whether the near approach of caries, traumatic injury, or thermal changes, devitalization usually gives present relief; it does not, however, always arrest the pathological changes due to pulp irritation. They may indeed be for the time arrested, but that does not insure the parts returning to a normal condition. In many cases the injury done is never fully repaired. We must remember the peculiar anatomical conditions here existing,—conditions found nowhere else in the human body. Within the soft tissues distending vessels meet with little resistance, the adjacent tissue permits increase of calibre and serous effusion with impunity; around the apex of the root, surrounded as it is by practically unyielding bony walls, this can take place only to a very limited extent without being accompanied by serious injury that will remain long after the cause producing it has ceased to exist.

It is a well-recognized sequence of inflammation that a part once so affected is peculiarly liable, upon slight and otherwise insufficient cause, to a return of that peculiar pathological condition and those other conditions which arise or proceed from it. It would seem as though the circulatory vessels affected, although relieved of the undue tension the disturbed circulation has produced, do not at once recover their normal tone. We may roughly express the idea by saying that the elastic or muscular coating has been stretched or strained beyond its normal elasticity, and but slowly, if ever, returns to a normal condition. The greater the degree of inflammation, or the longer time it has continued, the more slowly and the more uncertain is this return to normalcy.

Again, we may have within the tooth-pulp, without its having seriously disturbed the comfort or even having at any time attracted the patient's attention,—the result of caries, thermal changes, violence, or other causes,—a slowly progressive irritation that may painlessly originate, and may so continue for a long time; it may proceed so far that devitalization of the pulp takes place, and other progressive pathological changes follow one after another, until there is established a chronic alveolar abscess with well-defined fistulous opening, through which pus is being more or less freely discharged. These cases are by no means rare. It may happen that at any stage of such a case, perhaps before devitalization of the pulp has taken place, and after any operation other than intentional devitalization could be successfully performed, a sudden increase of irritation compels the patient to seek advice and relief. Can we at all times with certainty, in any such case, accurately diagnose the exact condition there existing,—how long the irritation has continued, or how far the injury has extended,—or accurately gauge the recuperative powers of nature so as to fix in our own minds how nearly the parts will, under judicious treatment, return to and resume a normal condition and a normal relation to the general system?

The history of such a case is practically unknown to us, and equally uncertain must be the final result of any operation we may attempt towards the preservation of the tooth. I might, but do not think it necessary to do so, refer to many other conditions of greater or less serious character that precede and handicap at the very beginning the success of this operation, and which introduce, in spite of all the care and skill we may bring to bear upon it, an element of uncertainty. In a vast majority of cases,—we may perhaps say in all,—where intentional devitalization of the dental pulp is necessary before filling a carious cavity, this irritated condition of the pulp is present to a greater or lesser degree. Where this is confined to the pulp, we may perhaps say, when it is, it is a matter of but little moment. I have no doubt that this is sometimes the case, and equally have no doubt that in many cases —cases where it is not suspected, and where there is no indication of it—there is present in the tissues beyond the apical foramen the result, or it may be the cause, of the pulp lesion,—a degree of irritation. I have no question that, independent—in a measure at least—of the treatment we may adopt, upon the presence or the absence of this primary irritation the success or failure of the operation largely depends.

Now, before proceeding further, let us try to fix in our own minds what we mean by "success," in treating teeth intentionally devitalized. What do we hope for; what do we strive for; and where draw the line between success and failure?

The ideal successful case is one in which, first, there has existed no lesions other than those which may be entirely relieved by devitalization and removal of the pulp; and, second, where, these operations having been accomplished without accident, the pulp removed, and the space occupied properly filled, nature has accepted the situation, the nutrient currents of the pulp have been so diverted that there is no undue strain upon the vessels concerned, and every portion of the tissue surrounding the apex of the tooth receives a sufficient supply. Where this is the case, the relation of the tooth to the general system has been but little disturbed; it may not now, perhaps, have the power to resist the encroachment of destructive agents to so great a degree as when the pulp was intact; we cannot consider it as valuable and as trustworthy as a vital tooth. It is more liable to fracture, if from no other cause than the necessary removal of a large portion of its substance in the effort to reach and properly fill the pulp-chamber and its appendages. There seems, however, developed in the course of a few years—in many cases, not in all—a change in the character of the dentine and also of the enamel. It becomes more brittle, and, where exposed to mastication, the edges of cavities and projecting portions of the tooth are apt to shatter and break. There seems also, after the lapse of considerable time, in many cases, a tendency to recurring decay more marked than in vital teeth of the same character. A devitalized tooth may, however, and frequently does for many years in spite of this, continue to perform its proper functions with comfort and satisfaction, and practically its usefulness may be but little impaired.

Of the many teeth intentionally devitalized the percentage of cases that are thus really successful must ever remain unknown. To determine this a well-kept case-book affords but little data. Thousands of devitalized teeth are to-day doing excellent and comfortable service, and may continue to do so for years, in which every element upon which success in treating such teeth is supposed to be based has been violated. Pulps have died under fillings,—in many cases the sequence of pulp-capping,—in some cases the tooth has darkened from infiltration of decomposed pulp-tissue, and yet, if the patient's testimony be accepted, there has been at no time the slightest discomfort; the change in the color of the tooth being the only observable indication of the change in its condition. These teeth are, of course, a constant menace. Nature simply tolerates them; they prove nothing beyond the fact that at times nature will tolerate a great deal with but little complaint. A slight change in the system or in the surrounding parts, a change in the patient's health or the local effects of a cold, may at any time set in motion pathological changes that will quickly result in an alveolar abscess; this exciting cause being absent, for a long series of years this may be held in abeyance, and the tooth remain as comfortable and as useful as its better-conditioned neighbors.

Precisely the same condition may follow the intentional devitalization of a dental pulp. An undiscovered lesion, an overlooked accident in the operation of devitalization and subsequent filling, or causes beyond the operator's knowledge or skill, may, in spite of all that he can possibly do, place the tooth in a condition quite as menacing as in the case above referred to; and yet it may be tolerated and remain comfortable for a variable time,—it may be months, it may be years,—ready, however, to give trouble at any moment. With this element of uncertainty constantly present, with the additional uncertainty of being unable in most cases to determine with any accuracy whether untoward results are due to an imperfect operation, defective methods, or a pre-existing condition which no skill can detect or overcome, any attempt to formulate the results of this operation must ever be received with caution and distrust.

After careful study, extended over many years, I feel a constantly increasing inability to fix upon any standard by which to judge the results of pulp treatment other than this: the patient comes to us in distress, we hope by our treatment to afford relief, and strive to preserve the comfortable usefulness of the tooth. If we succeed in doing this, we may count the operation a success; only so long, however, as this comfortable usefulness continues. We must ever, in making up the record, remember that the result, favorable or unfavorable, may be as largely due to matters over which we have no control as to our own judgment and skill. The lapse of time, of itself, is no assurance of success. An intentionally devitalized tooth, imperfectly treated, is quite as likely to remain comfortable for a long time as is a tooth in which the pulp has died and has received no treatment. An intentionally devitalized tooth, which has received the best, most careful, and thorough treatment, may quickly give trouble from causes over which the operator has no control, and for which he is in nowise responsible.

I do not wish to be understood as intimating that treatment has but little to do with the result. In some cases it has not; in a large majority, however, judicious and thorough treatment will be followed by correspondingly good results.

I wish to state most emphatically my conviction that, under the most favorable conditions, a devitalized tooth is never as reliable and trustworthy as is a normally vital one. Accident or pathological conditions frequently make the surgeon's knife a necessity; not infrequently the surgeon is called upon to decide whether to sacrifice a limb or risk the patient's life. No matter how skilfully the operation is performed, or how quickly or thoroughly the patient recovers, or how perfectly the lost member is supplemented by dexterous use of those that remain, the patient is none the less a mutilated being, and to that extent his life and well-being is more frequently in jeopardy. A devitalized tooth is a mutilated tooth. The pulp may have, as some contend, ceased to be useful; I do not so think: so have some other organs of the body; notwithstanding that, however, their surgical removal would be attended by most serious results. So is—to the tooth at least, in many cases—the removal of the tooth-pulp from precisely the same cause: not so much the loss of the organ as the injury necessarily done to adjacent parts in effecting its removal.

Various attempts have been made to so modify the operation of devitalizing a dental pulp as to lessen the risk of after trouble. Arsenic, the agent usually employed, has been charged—unjustly, I think—with causing many of these evils. In careless hands, we all know it is capable of doing a great deal of mischief; but we are not considering that now. We are presuming that the operation from beginning to end is carefully and skilfully performed. It has been asserted that the arsenic may escape through the apical foramina, or in the same way that it acts upon the pulp, without really passing through this opening; it may, and in many cases does, produce its peculiar effect upon the tissues at the apex of the root, and thus lay the foundation for future trouble. To avoid this, extremely small quantities have been recommended, or it is left in contact with the tissue but a short time. How arsenic effects the destruction of a dental pulp is, I think, an unsolved problem. We are taught that it is absorbed by living tissue only, and when applied as an escharotic for the removal of tumor, etc., its action is limited by using it freely, so that it quickly destroys the tissue with which it is in immediate contact; this dead tissue then acts as a barrier protecting the living tissue beyond. In these cases, were it applied less freely, it is quite probable that so much would be absorbed that serious systemic effects would follow. Many years ago, when it was recommended and used for treating sensitive dentine, it was soon found that no precaution prevented the complete devitalization of the pulp; it was utterly impossible to limit its action to the dentine alone. Even when the arsenic was finely pulverized, used dry, in its least active form, and for a few hours only sealed up in a superficial cavity, the tooth-tissue, immediately after its removal, being excavated so deeply that every portion with which the arsenic had been in contact was removed invariably; notwithstanding all these precautions, the complete devitalization of the pulp was a question of time only. This being the case, how absurd the idea that by pricking into the exposed portion of the pulp a minute quantity of arsenic a portion only of the pulp will be destroyed and the apical one-third or two-thirds remain vital! I cannot conceive this to be possible in any case. I know that on attempting to remove a devitalized pulp we may find the apical portion quite sensitive to the touch of the instrument; I know, also, that in teeth with more than one root the pulp may readily and without pain be removed from one while its removal from the others—usually the smaller roots—may be painful and difficult; but is this any evidence of vitality? We frequently find the same sensitiveness upon first opening into an intentionally devitalized pulp,—so sensitive are they, occasionally, that it is hard to realize that the arsenic has done its work; and yet, after thoroughly exposing the pulp, there has been no difficulty in effecting its complete removal painlessly, showing conclusively that it had been completely devitalized. What reason have we to suppose that the sensitiveness in one case indicates vitality, when we know that in the other, precisely similar, it does not?

To avoid the evils supposed to be associated with the use of arsenic other means of devitalization have been employed; I presume that they are all perfectly familiar to you. Believing, as I do, that they possess no advantage over the use of arsenic, I do not propose to consider them. To my mind it is not the means employed: it is not so much—although much may depend upon it —the after-treatment the tooth may receive, the local changes made necessary by the devitalization of the pulp; the local changes that may have preceded and those that necessarily follow the operation are the real enemies with which we have to contend.

Recall, if you please, the general anatomy of the dental pulp. When this is devitalized and no longer able to utilize the nutrient currents that have been flowing to and from it, what takes place? First, with the nutrient vessels there is a stagnation of their contents; at some point these vessels become permanently closed; then the nutrient currents, no longer able to follow the accustomed channels, seek other openings; an anastomosis, as it is termed, takes place. Smaller vessels are enlarged, and in a short time the current that once flowed into the pulp now flows by it. It is now very possible that, as a result of this change, portions of the tissues around the apex of the root, formerly supplied by vessels branching from the occluded portions of the arteries and veins leading to and from the pulp, may be deprived in a measure of their nutrient supply, and thus predisposed to break down from slight systemic or local causes. May not this explain the origin of alveolar abscess that has no connection with, and cannot be reached through, the apex of the root, and of abscess that occurs for the first time many years after devitalization and treatment of the pulp?

I appreciate fully how important and useful have been the results of the close study of the cause of traumatic irritation during the last few years. It has enabled us to work with more confidence, and to obtain better results in many cases. It has not, however, given us much assistance in treating intentional devitalization. In uncomplicated cases, where the pulp can be thoroughly removed and the space it occupied as thoroughly filled, there is little need for antiseptics. In other cases, where, on account of the position of the cavity, condition of the pulp-canals, or restlessness of the patient, thorough removal becomes impossible, we are compelled to trust more to the kindliness of nature than to the resources of art. I question seriously the real practical value of antiseptics in such cases. If the tissues we desire to render inert were perfectly dry, or in a condition to readily absorb and become thoroughly impregnated with the antiseptic agent, it would undoubtedly accomplish its object; but such is not the case, nor can they be so made. The application of heat, by means of dry, heated atmospheric air or a heated instrument, is so limited by the sensitiveness of the parts that the effect must be superficial and of questionable usefulness.

The named methods of treatment have been so thoroughly written upon and discussed that I can say but little upon this subject that you do not already know. From the fact that advocates of widely-differing methods claim each for his own a very satisfactory measure of success, I have come to regard it largely a matter of individual preference. The same may be said of the material used for root filling. Provided the tooth remains as we leave it, there is little to choose among the many available methods recommended for this purpose. In case decay should recur and extend so as to again reach the pulp cavity, an indestructible material like gutta-percha, tin, lead, gold, etc., provided it makes a perfect filling, would then have a very decided advantage over others that readily disintegrate.

In conclusion, I advocate strongly the conservation of the dental pulp in all cases where it can be attempted with a fair chance of success. I recognize at the same time that in many cases the devitalization of the pulp, with all its attendant evils, is decidedly the best course to pursue. We will not always attain desired success. We must expect some failures from causes beyond control. We will have occasional irritation, occasional abscess, and occasional extraction.

